# A Single Nucleotide Polymorphism Is Involved in Regulation of Growth and Spore Formation of *Bacillus anthracis* Pasteur II Strain

**DOI:** 10.3389/fcimb.2017.00270

**Published:** 2017-06-28

**Authors:** Xudong Liang, Jin Zhu, Zhongzhi Zhao, Feng Zheng, Enmin Zhang, Jianchun Wei, Yon Ji, Yinduo Ji

**Affiliations:** ^1^State Key Laboratory for Infectious Disease Prevention and Control, Chinese Center for Disease Control and Prevention, National Institute for Communicable Disease Control and PreventionBeijing, China; ^2^Huadong Medical Institute of BiotechniquesNanjing, China; ^3^Department of Veterinary and Biomedical Sciences, College of Veterinary Medicine, University of Minnesota, St. PaulMN, United States

**Keywords:** *Bacillus anthracis*, single nucleotide polymorphisms (SNPs), regulation, bacterial growth, spore formation

## Abstract

Anthrax toxins and capsules, which are encoded by genes located on pXO1 and pXO2, respectively, are major virulence factors of *Bacillus anthracis*. Our previous studies demonstrated that exposure to high-temperatures is unable to abolish the pXO1 plasmid of the Pasteur II strain, but the growth of the strain was obviously slower than that of the Sterne strain and wild-type virulent strain. To elucidate a potential regulatory mechanism of slowing growth, we employed comparative genome and bioinformatic analysis and revealed a unique SNP (G to T) at the 143135 bp position in pXO1 that is possibly involved in the mediation of growth of Pasteur II. However, the T to G mutation in *groR* did not result in any change of the amino acid sequence. A predominant nucleotide G existed at the 143135 bp in pXO1 of 100 wild-type *B. anthracis* isolates and 9 isolates documented in GenBank, whereas T replaced G in pXO1 of the Pasteur II strain. Further analysis indicate that the SNP is located in a gene between 143042 and 143173 bp, and that it encodes a small protein of 43 amino acids and is termed as a growth regulator (GroR). Site-directed mutagenesis and gene deletion demonstrates that *groR* regulates the growth and spore formation of *B. anthracis*. Our results indicate that the pXO1 plasmid is involved in the regulation of growth and spore formation in *B. anthracis*.

## Introduction

*Bacillus anthracis* is a spore-forming, gram-positive bacterium and it exists as dormant spores in the environment. Spores are highly resistant to temperature changes, thus enabling *B. anthracis* to survive for long periods of time. When the spores enter a mammalian host through a cutaneous injury, gastrointestinal ingestion or inhalation, the bacteria rapidly proliferate and produce anthrax toxins and capsule (Liang, 1995).

The anthrax toxins are encoded by *cya, lef*, and *pag*A *genes*, which are located in a pathogenicity island of pXO1 (Mikesell et al., 1983; Okinaka et al., 1999). The production of pXO1 virulence factors is regulated by the pXO1 transcriptional regulator AtxA (Uchida et al., 1993, 1997; Koehler et al., 1994; Hoffmaster and Koehler, 1997), carbon dioxide, and a bicarbonate condition that occurs upon transition of the pathogen from the external environment to the host (Ezzell and Abshire, 1988; Bartkus and Leppla, 1989; Koehler et al., 1994; Sirard et al., 1994). The poly-r-D-glutamic acid capsule is synthesized by the *capBCADE* operon located on pXO2 (Uchida et al., 1986; Makino et al., 1989).

The genetic regulation of *B. anthracis* virulence factors and the pathogenic mechanism have been well documented. It was reported that *gne*Z is required for *B. anthracis* vegetative growth, rod cell shape maintenance, S-layer assembly, and synthesis of pyruvylated secondary cell wall polysaccharide (Wang et al., 2014). *B. anthracis* is dependent on siderophore biosynthesis for survival in macrophages. The single-walled carbon nanotubes affect *B. anthracis* cell growth, spore formation, and spore germination (Cendrowski et al., 2004; Aferchich et al., 2012). The germination-specific lytic enzymes, including SleB that is regulated by YpeB, are necessary for spore germination (Bernhards et al., 2015). HtrC is a protease responsible for specific cleavage and generation of a stable YpeB (Bernhards et al., 2015), however, HtrC had no impact on the stability of YpeB or SleB during spore formation in the absence of the partner protein in *B. anthracis* and *Bacillus subtilis* spores, indicating that other proteases are involved in their degradation during sporulation. Moreover, inactivating *sleL*, which encodes a cortex-lytic enzyme, did not alter vegetative growth, spore viability, or the initial stages of germination, including dipicolinic acid release (Lambert and Popham, 2008; Bernhards and Popham, 2014; Bernhards et al., 2015).

Our previous studies demonstrated that high temperature treatment was unable to completely eliminate the pXO1 plasmid of the vaccine Pasteur II strain, although the growth of the strain was obviously slower than that of other strains, including the Sterne vaccine strain and wild-type virulent *B. anthracis* (Liang et al., 2016). The aim of this study was to elucidate the mechanisms involved in regulating the growth of the Pasteur II strain. We performed a comparative genome and bioinformatic analysis of Pasteur II against the remaining *B. anthracis* genomes and found a unique SNP (G to T) at 143135bp at the pathogenicity island of pXO1 in the Pasteur II strain. We revealed that the T-G SNP is located between two genes *pagA* and *ger*X, which is involved in the virulence of *B. anthracis* (Guidi-Rontani et al., 1999). This mutation occurred in a small protein-coding region near the *pag* gene promoter region. To determine whether the SNP contributes to the regulation of growth, we performed site-directed mutagenesis, gene deletion, and complementation studies. Our data collectively demonstrated that the pXO1 plasmid plays an important role in regulation of *B. anthracis* growth and spore formation.

## Materials and methods

### Bacterial vaccine strains

*B. anthracis* vaccine Pasteur II strains were provided by the Institute of Lanzhou Biological Products in China (Liang et al., 2016).

### PCR and DNA sequencing of single nucleotide polymorphisms (SNPs) regions

The oligo primers were designed by using upstream Oligo 6.0 software PCR regions containing SNP at site 143135 bp. The upstream primer was PAsnpF: 5′-TCAAACCACCTAACAAACAGC-3′, the downstream primer was PAsnpR: 5′-GTTTGTTTTTTTATTATTTTTTCTA-3′. PCR was performed using High fidelity polymerase PyrobestTaq (TaKaRa Biotech Co. Ltd., Dalian, China) using the following program: initial denaturation at 95°C for 5 min, followed by 30 cycles of denaturation (95°C for 30 s), annealing (55°C for 30 s), and extension (72°C for 1 min). The size of the expected amplicon was 765 bp. The purified PCR products were sequenced using Sanger Double Deoxidizing Chain Termination Method and the sequences were utilized for comparative genome analysis by BLAST against the published genomes in NCBI genome database using Mega 6.0.

### Construction of the SNP site-directed mutation strain

Primers were designed, according to the sequence of plasmid pXO1 covering 400 bp upstream and downstream from the target site (T-G), using Oligo 6.0 software and the sequences were PA-promMUT-F: 5′-GGAGGATCCTCAAACCACCTAACAAAC AGC-3′ and PA-promMUT-R: 5′-AGAAGATCTGTTTGTTTTTTTATTATTTTTT CTA-3′. A *Bam*HI site was introduced into the upstream primer and a *Bgl*II site was created in the downstream primer. A fragment of 783 bp in length was amplified by PCR using wild-type strain DNA as a template. The ligated products of the PCR amplicons and plasmid pMAD, which were both double digested with *Bam*HI and *Bgl*II, were transformed into *E. coli*. The resulting recombinant plasmid T-G-pMAD was transformed into Pasteur II by electroporation through SCS110. The bacteria integrated with T-G-pMAD in the genome were harvested at 42°C and then subcultured under low temperature to screen for erythromycin-sensitive colonies. The gene-replacement strain, designated Pasteur II-T/G, was identified using PCR and sequence analysis.

### Construction of the growth regulator gene (*groR*) deletion strain

Overlap-PCR was used to amplify the homologous region located upstream and downstream of the deletion region. The primers directed to the upstream regulation of growth gene region were: 43aaKOupF/BamHI: 5′-GCTCGGATCCTAATAACGGT AATATTGTAGGG-3′, and 43aaKOcrossover: 5′-ATACTGGTTAAATTCACAAT AACTACAACTGTCCAAGCTAAC-3′, with a *Bam*HI site designed into the forward primer. An upstream homology arm ~1,000 bp was amplified by regular PCR with wild-type strain DNA as template. The downstream primers were 43aaKOcrossoverF: 5′-GTTAGCTTGGACAGTTGTAGTTATTGTGAATTTAACCAGTAT-3′, and 43aaKOdnR/Bgl: 5′-CGGGAGATCTGATAAATCCTGACCAAATAGC-3′) with a *Bgl*II designed into the reverse primer. A downstream homology arm ~800 bp was amplified. Since parts of the sequences of 43aaKOcrossoverR and 43aaKOcrossoverF3 were complementary to each other, the final fragment homologous to the upstream and downstream of the deletion region was amplified by PCR using the primers 43aaKOupF/BamHI and 43aaKOdnR/BglII, digested with *Bam*HI and *Bgl*II, then cloned into the pMAD plasmid, which was then digested using the same enzymes to produce the recombinant plasmid pMAD43aaKO. The recombinant plasmid was activated using SCS110 and electroporated into the Pasteur II strain. The growth regulation gene (GroR) deletion strain designated Pasteur II—GroRKO was identified from all erythromycin sensitive strains using PCR and sequencing confirmation.

### Construction of the GroR gene complementary strain

Primers directed to GR gene were designed using Oligo 6.0 software (43aaOESacF:5′-CAGCCGCGGATGAAACAAAGATTATTTAGGTT-3′ and 43aaOESacR:5′-CAGCCGCGGTTATAACGTATAAAATTTCACGCAC-3′). High-fidelity polymerase Pyrobest (TaKaRa Biotech Co. Ltd., Daliang, China) was used to amplify the target gene fragment on the pXO1 plasmid. After initial denaturation at 95°C for 5 min, the reaction was carried out for 30 cycles, each consisting of denaturation (95°C for 30 s), annealing (55°C for 30 s), and extension (72°C for 30 s). A 129 bp fragment was amplified and purified, followed by digestion with *Sac*II enzyme, and then cloned into the expression vector pFF40 digested by *Sac*II. The recombinant plasmid was transformed into *E. coli* DH5α, and the positive clone was screened by PCR and identified by double digestion. After the plasmid was electroporated into the Pasteur II-GroRKO strain and selected using kanamycin, the positive strain was further confirmed using DNA sequencing to ensure there was no mutation in the protein. The positive clone was designated as the Pasteur II-GroRKO-CO strain.

### Characterization of bacterial growth

Cultures of *B. anthracis* strains grown overnight in Lysogeny Broth (LB) were diluted in fresh medium to an OD600 of 0.08. Bacterial cells covered with oil were cultured in Falcon tubes (50 ml) with moderate shaking (200 rpm). Cell growth at 37°C was monitored by measuring the optical density at 600 nm using a SpectraMax Plus spectrophotometer. Triplicate readings were obtained at different times during growth.

### The schaeffer-fulton endospore staining

All stains were stored at room temperature. Aqueous malachite green (5%) and crystal violet solution (5%) (Yinqiang Co., Beijing) was used for endospore staining using the Schaeffer-Fulton endospore stain protocol, with the following modification: the 5% aqueous malachite green primary stain was retained; however, a 5% crystal violet solution was used as the counterstain.

### Western blot analysis

We assessed spore formation by immunoblot analyses using anti-Spores antibody. The Pasteur II strain and targeted knockout strain were cultured on LB plates at 37°C for 24–48 h. The bacterial cultures were collected and suspended in 150 μl PBS buffer. The whole bacterial proteins were prepared by mixing with SDS-PAGE loading buffer followed by boiling at 100°C for 10 min. Twenty microliters of each sample was loaded onto a 12% SDS-PAGE gel. The proteins were then transferred onto a nitrocellulose membrane (Bio-Rad, Hercules, CA, USA). After being blocked with PBS containing 5% skim milk, the membrane was incubated with anti-Spores antibody (Thermo Scientific) at a 1:500 dilution or anti-protective antigen (PA) antibody (Thermo Scientific) diluted 1:2,000 in TBS in 5% milk overnight at 4°C. After being washed 3 times with PBST, the membrane was incubated with a secondary HRP-conjugated Goat anti-Mouse antibody at 1:4,000 dilutions for 1 h at RT. After being washed, the proteins were detected using the ECL Western Blot Substrate (Pierce) Kit according to the manufacturer's instruction. The density of the bands on the membranes was analyzed using ImageJ Software, and the values of the density were average from three independent experiments. The difference was statistically analyzed using Student T test.

## Results

### Identification of single nucleotide polymorphism (SNP) (G-T) at the 143135bp position of pXO1 in Pasteur II vaccine strain

**Figure 1:**
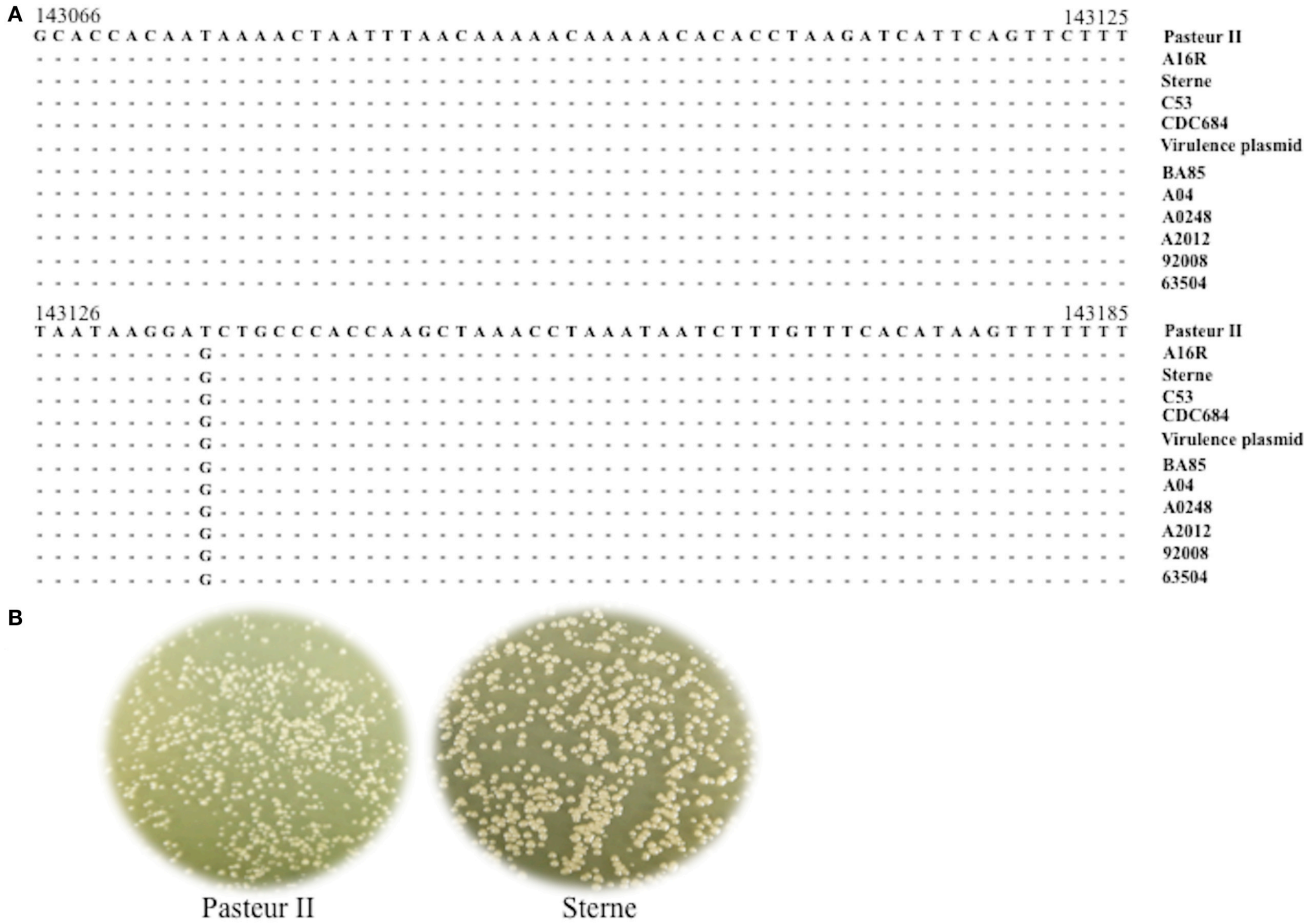
Identification of SNP in pXO1 of *B. anthracis*. **(A)** Structural gene sequence alignment of a SNP region in pXO1. **(B)** Morphology of bacteria colonies. Pasteur II and Sterne was plated onto LB-agar plate for incubated overnight at 37°C. The images are representative of three independent experiments.

To elucidate the potential mechanisms involved in down-regulating the growth of the Pasteur II strain, we performed DNA sequence alignment analyses of the pXO1 plasmid whole sequence, based on the published *B. anthracis* genome sequences in the NCBI genome database. We found a unique SNP, T, at the 143135 bp position of Pasteur II strain compared to the rest of the strains listed in Figure 1. To further investigate whether this difference exists in other *B. anthracis* isolates, we isolated the chromosomal DNA from 100 wild strains, and amplified and sequenced the DNA regions covering the SNP position. The results showed that nucleotide G, at the corresponding position of Pasteur II, was present in all tested wild-type strains, whereas nucleotide T was only present in the Pasteur II strain (Figure 1A). The Pasteur II strain showed smaller colonies than Sterne strain (Figure 1B). Further analysis revealed that the SNP was located in an open reading frame that encodes a small protein composed of 43 amino acids, which was designated as a growth regulator (GroR). The *groR* gene was located upstream of the promoter region of the *pagA* gene that encodes the *B. anthracis* protective antigen.

### The G to T mutation at the 143135bp position of pXO1 inhibits bacterial growth

**Figure 2:**
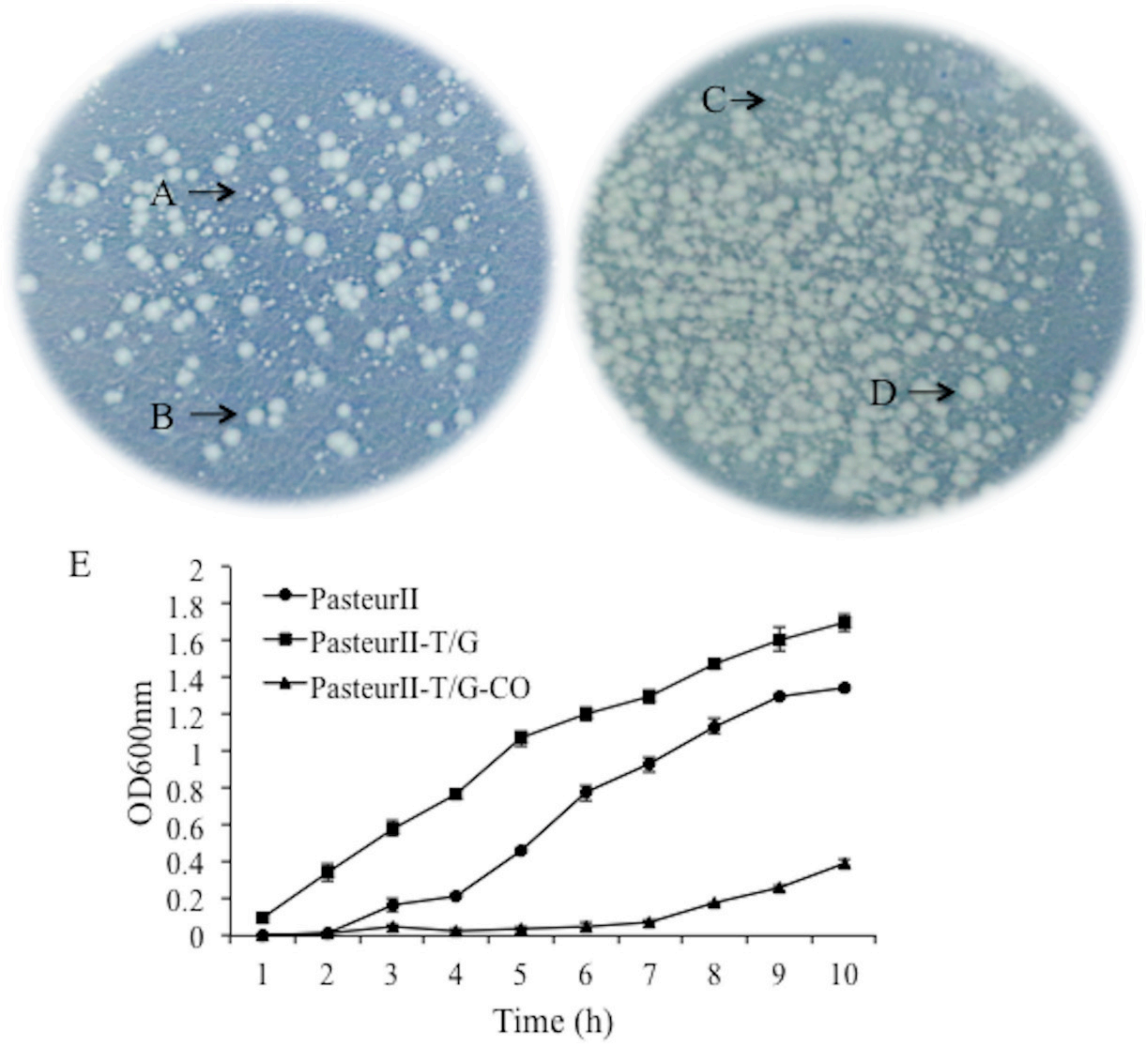
Morphology of bacteria colonies. The mix-culture of the T-G site mutant and its parental control, Pasteur II, was plated onto LB-agar plate for viable CFU and incubated overnight at 37°C. **(A)** is Pasteur II strain, **(B)** is Pasteur II-T/G site mutation strain, **(C)** is PasteurII, and **(D)** is PasteurII-GroRKO mutation strain. The images are representative of three independent experiments. **(E)** Effect of the T-G mutation on bacterial growth.

The identification of a specific SNP (G to T) at the 143135 bp position of pXO1 of Pasteur II strain led us to hypothesize that this SNP may be associated with slower growth. To test this possibility, we first created a T-G site-directed mutation strain using a homologous recombination. The nucleotide T at 143135 bp on the pXO1 plasmid of Pasteur II vaccine strain was changed into G to form the strain Pasteur II-T/G. Then, we determined the effect of the T-G site mutation on the phenotypes of bacterial growth. After mix-culturing the mutant and the control strain under the same conditions on the solid LB-agar plate, two different sizes of colonies were obtained; and the colonies of the T-G site mutant strain (Figure 2B) were larger than those of the original Pasteur II strain (Figures 2A,C). Moreover, the colonies with the T-G site mutation exhibited a rougher morphology, with irregular edges, as compared to the parental control (Figure 2).

### The short ORF between 143042 to 143173 bp in pXO1 termed *groR* affects growth and spore formation

**Figure 3:**
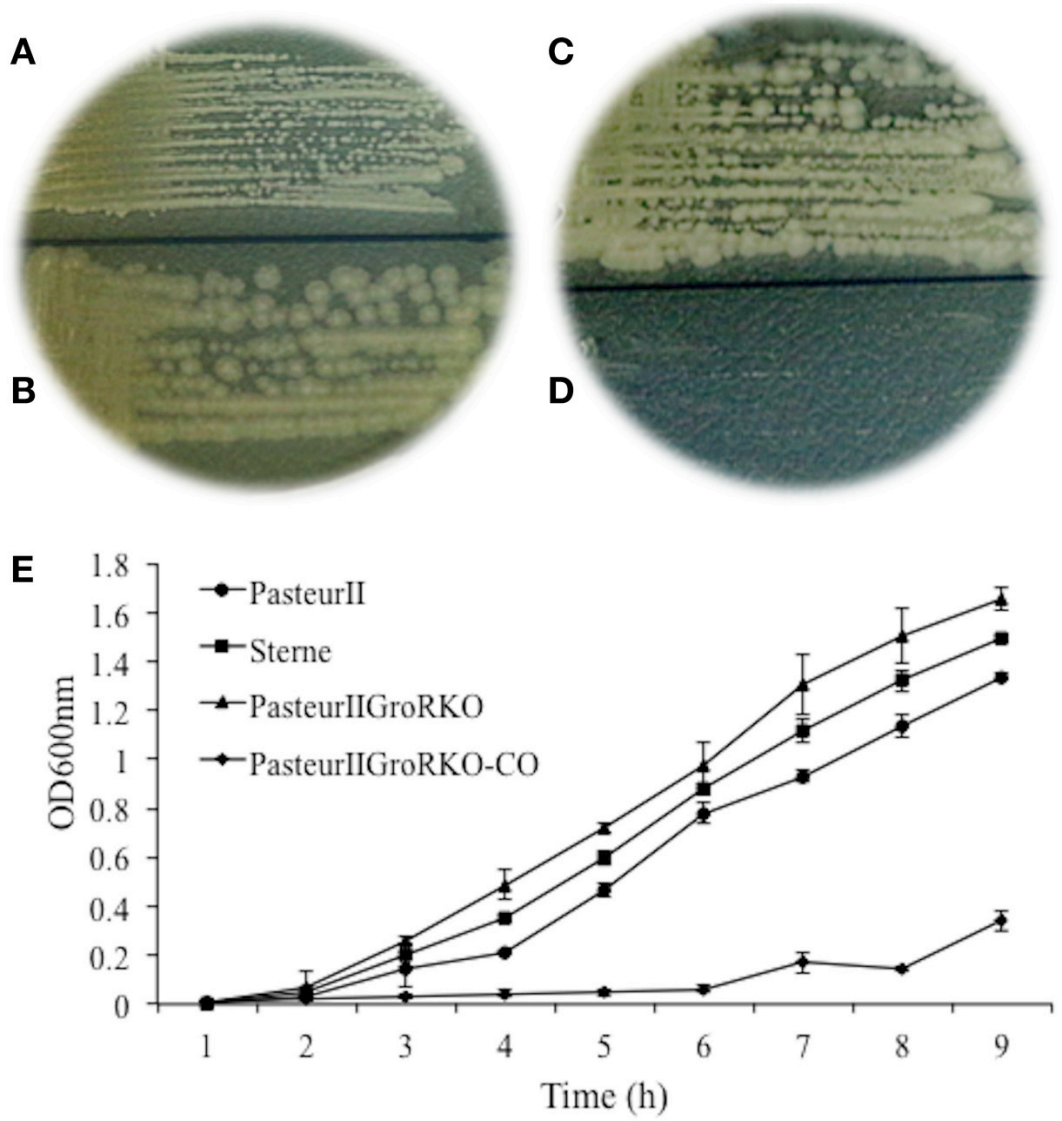
**(A–D)** Morphology of bacteria colonies. The culture of the T-G site mutant, Pasteur II GroKO, and its parental control, Pasteur II, were streaked onto LB-agar plate, respectively, for viable CFU and incubated overnight at 37°C. **(A)** is Pasteur II strain, **(B)** is Pasteur II -GroRKO strain, **(C)** is Pasteur II -GroRKO strain, **(D)** is Pasteur II -GroRKO-CO strain. **(E)** Growth curves of Pasteur II, Pasteur II-GroRKO, and Pasteur II-GRKO-CO. The images are representative of three independent experiments.

The unique SNP at 143135 bp was located in a short ORF from 143042 143173 bp, which encodes a small protein of 43 amino acids and is designated as GroR. To determine the role of this short ORF in growth, we constructed a *groR* null deletion mutant and examined the effect of *groR* deletion mutation on the colony size on a solid medium and growth in a liquid medium. The results showed that the colony size and growth rate of the II-GroRKO strain were larger (Figures 3A–D) and faster (Figure 3E), respectively, than those of the parental Pasteur II strain. We designated this ORF as a novel growth regulatory gene, *groR*, because of its involvement in bacterial growth.

**Figure 4:**
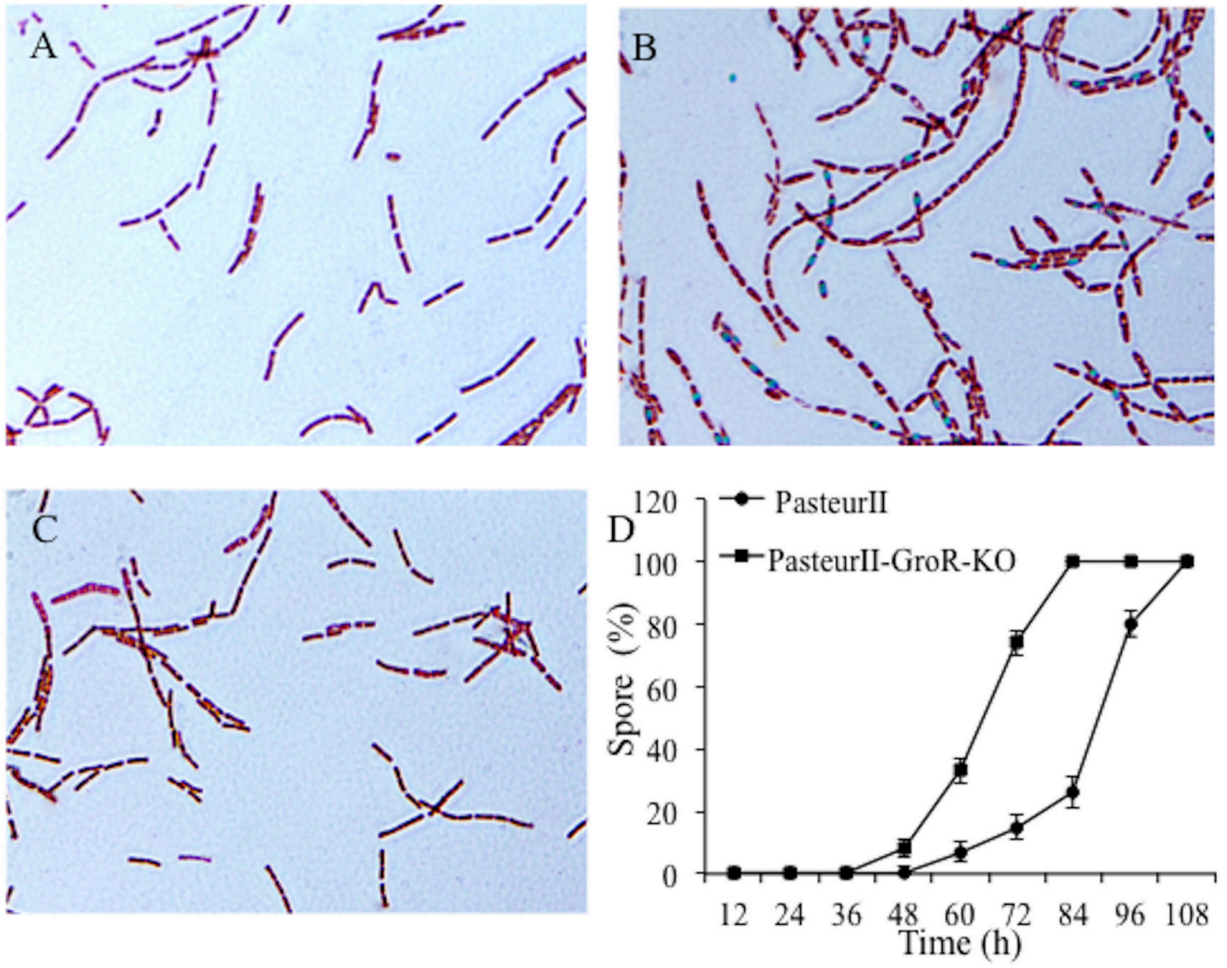
Effect of GroR on spore formation. The *groR* null mutant and its parental control grew in LB medium for 24 at 37°C. The bacterial cells were smeared, stained, and observed under microscope. **(A)** is Pasteur II strain, 24 h, **(B)** is Pasteur II -GroRKO strain, 24 h. The images are representative of three independent experiments. **(C)** is the Pasteur II-GroRKO-CO strain. **(D)** Quantitative analysis of rate of cells to form spores with heat-treatment of culture.

To examine the impact of *groR* on spore formation, bacterial cells were cultured for 24 and 48 h, stained on smears, and observed under a microscope. Interestingly, cells of the Pasteur II -GroRKO strain were bulky in size and some had already formed spores, while its parental control, the Pasteur II strain, had no spore formation at 24 h (Figure 4). The number of cells that formed spores at 36 h was 70% for the Pasteur II -GroRKO strain and 30% for the Pasteur II strain.

**Figure 5:**
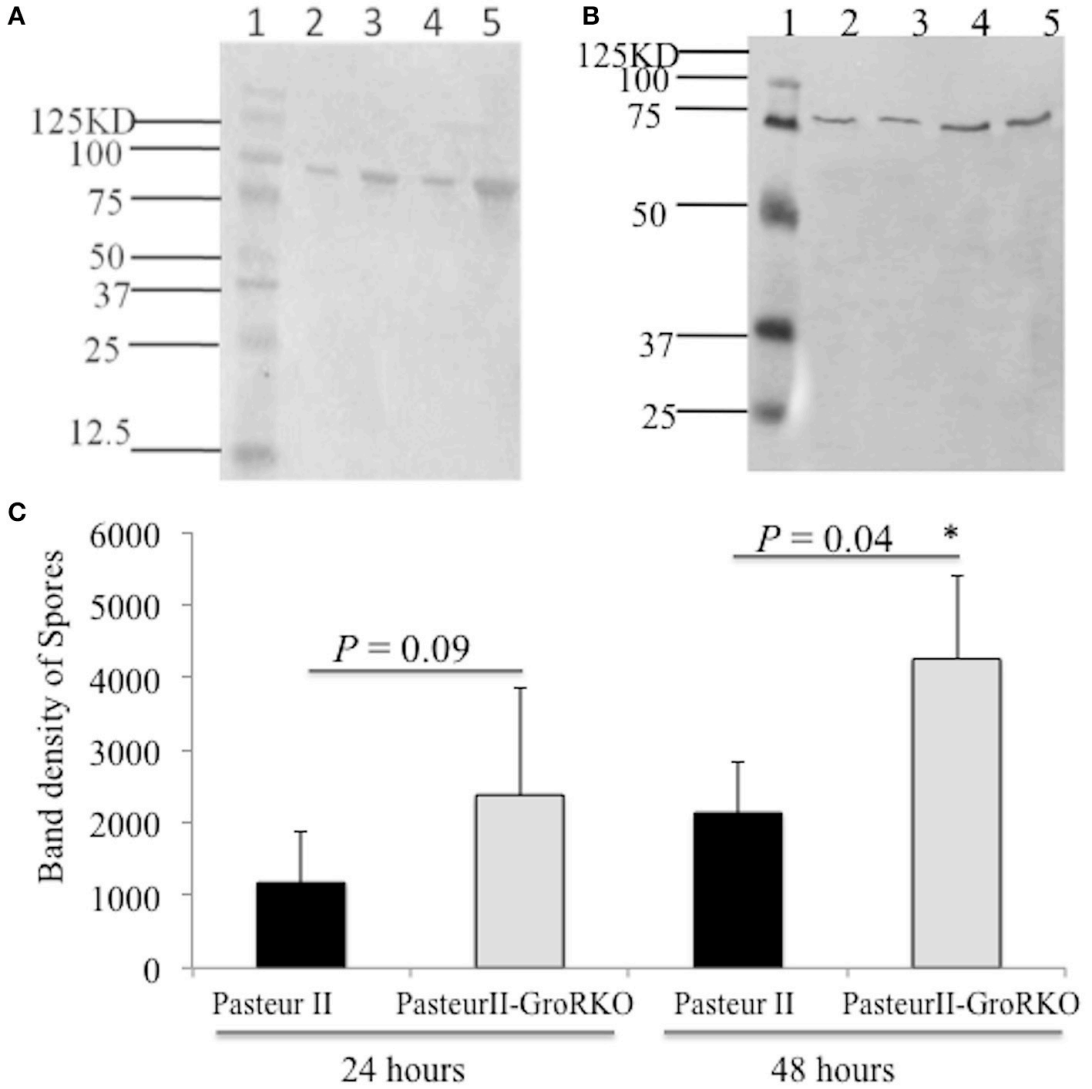
Western blotting detection of spores formation in variant strains. **(A)** is anti-Spores antibody; **(B)** is a control with anti-protective antigen (PA) antibody. Lane 1 is Protein Molecular Weight Marker, Lane 2 is Pasteur II strain, 24 h, Lane 3 is PasteurII-GroRKO strain, 24 h, Lane 4 is Pasteur II strain, 48 h, Lane 5 is PasteurII-GroRKO strain, 48 h. **(C)** is quantitative analysis of the spore formation. The relative density of the band on the membrane was obtained by using ImageJ software and statistically analyzed. ^*^P < 0.05, significant difference.

To further confirm the effect of GroR on spore formation, we performed Western-blotting assays to examine the expression of spore proteins. As shown in Figure 5A, the PasteurII-GroRKO strain produced many more spore proteins than its parental control, the Pasteur II strain, after 24 and 48 h of incubation, respectively. Moreover, the deletion mutation of *groR* had no effect on the expression of *pag* (Figure 5B), whereas significantly enhanced the production of spore proteins after 48 h of incubation (Figure 5C).

## Discussion

In this study, we investigated the molecular mechanism underlying the slower growth of the Pasteur II strain compared to other vaccine strains such as the Sterne strain and wild-type *B. anthracis*. A remarkable difference of the colony size and the lag phase was observed between Pasteur II and Sterne strains (Figures 1B, 3E). It is possible that due to the lack of energy and necessary growth factors in the new conditions, a longer adjustment period was required for the Pasteur II strain compared to the other strains. Our studies revealed that the region near the *pag* gene promoter harbors a predominant SNP that contributes to the regulation of the growth and spore formation in *B. anthracis*. We discovered that the unique T-G SNP in pXO1 involved in the regulation of *B. anthracis* growth. The SNP formation in Pasteur II strain is likely attributable to high temperature stress, which affects toxicity of *B. anthracis*, but has no impact on spore resistance to high temperature treatment. However, there was no G to T mutation in other *B. anthracis* strains.

It has been observed that *B. cereus* (NCTC 8234) grows much faster and forms larger colonies than the *B. anthracis* Sterne strain. The major difference between these two strains is that *B. anthracis* carries the pXO1 plasmid, whereas there is no pXO1 in *B. cereus* (Ash et al., 1991; Ash and Collins, 1992; Liang et al., 2016). In this study we demonstrated the role of pXO1 plasmid in modulation of bacterial growth and spore formation, which could explain why *B. anthracis* Sterne strains grow slower than *B. cereus*.

Using site-directed mutagenesis and gene knockout approaches, we demonstrated that the pXO1 plasmid also influences the spore formation of *B. anthracis*. It was reported that many genes are involved in regulation of spore formation of *B. subtilis* and *B. anthracis*. Guidi-Rontani et al. have identified a cluster of germination genes extending for 3608 nucleotides between the *pag* and *atx*A genes on the *B. anthracis* virulence plasmid pXO1. We discovered the unique T-G SNP between two genes *pagA* and *ger*X on the pXO1 plasmid, and concluded that this SNP regulated bacterial growth and spore formation of *B. anthracis*.

Based upon the DNA sequences of the SNP region, we found a novel open reading frame (gene) in pXO1, which is designated as *groR* due to its function in repression of *B. anthracis* growth. However, the T to G mutation in *groR* did not result in any change of the amino acid sequence, and overexpression of GroR in the complementary strain, Pasteur II GroRKO-CO, suppressed the growth. Taken together, these observations suggest tht *groR* may function using an alternative mechanism such as a microRNA, which needs to be further investigated. The unique T-G SNP may alter the microRNA structure to interfere with its binding to target mRNA. This may consequently inhibit the growth of *B. anthracis* vaccine Pasteur II strain.

In conclusion, we are the first to demonstrate that the pXO1 plasmid plays an important role in repression of growth and spore formation in *B. anthracis* Pasteur II strain. Particularly, we have identified and demonstrated that the T-G SNP in *groR* between 143042 and 143173 bp on pXO1 is responsible for regulation of Pasteur II strain growth. This finding provides a new insight into the role of pXO1 in *B. anthracis* Pasteur II.

## Author contributions

Conceived and designed the experiments: XL. Performed the experiments: XL, ZZ, FZ, EZ. Analyzed the data: XL, JZ, JW, YoJ, YiJ. Wrote the paper: XL, YiJ.

### Conflict of interest statement

The authors declare that the research was conducted in the absence of any commercial or financial relationships that could be construed as a potential conflict of interest.
